# Ischemic Stroke in Non-Gender-Related CHA_2_DS_2_-VA Score 0~1 Is Associated With H_2_FPEF Score Among the Patients With Atrial Fibrillation

**DOI:** 10.3389/fcvm.2021.791112

**Published:** 2022-02-08

**Authors:** Min Kim, Hee Tae Yu, Tae-Hoon Kim, Dae-In Lee, Jae-Sun Uhm, Young Dae Kim, Hyo Suk Nam, Boyoung Joung, Moon-Hyoung Lee, Ji Hoe Heo, Hui-Nam Pak

**Affiliations:** ^1^Division of Cardiology, Chungbuk National University Hospital, Cheongju, South Korea; ^2^Division of Cardiology, Yonsei University Health System, Seoul, South Korea; ^3^Department of Neurology, Yonsei University Health System, Seoul, South Korea

**Keywords:** atrial fibrillation, CHA_2_DS_2_-VA score, H_2_FPEF score, stroke, atrial myopathy

## Abstract

**Background:**

Ischemic strokes (ISs) can appear even in non-gender-related CHA_2_DS_2_-VA scores 0~1 patients with atrial fibrillation (AF). We explored the determinants associated with IS development among the patients with non-gender-related CHA_2_DS_2_-VA score 0~1 AF.

**Methods and Results:**

In this single-center retrospective registry data for AF catheter ablation (AFCA), we included 1,353 patients with AF (24.7% female, median age 56 years, and paroxysmal AF 72.6%) who had non-gender-related CHA_2_DS_2_-VA score 0~1, normal left ventricular (LV) systolic function, and available H_2_FPEF score. Among those patients, 113 experienced IS despite a non-gender-related CHA_2_DS_2_-VA score of 0~1. All included patients underwent AFCA, and we evaluated the associated factors with IS in non-gender-related CHA_2_DS_2_-VA score 0~1 AF. Patients with ISs in this study had a lower estimated glomerular filtration rate (eGFR) (*p* < 0.001) and LV ejection fraction (LVEF; *p* = 0.017), larger LA diameter (*p* < 0.001), reduced LA appendage peak velocity (*p* < 0.001), and a higher baseline H_2_FPEF score (*p* = 0.018) relative to those without ISs. Age [odds ratio (OR) 1.11 (1.07–1.17), *p* < 0.001, Model 1] and H_2_FPEF score as continuous [*OR* 1.31 (1.03–1.67), *p* = 0.028, Model 2] variable were independently associated with ISs by multivariate analysis. Moreover, the eGFR was independently associated with IS at low CHA_2_DS_2_-VA scores in both Models 1 and 2. AF recurrence was significantly higher in patients with IS (log-rank *p* < 0.001) but not in those with high H_2_FPEF scores (log-rank *p* = 0.079), respectively.

**Conclusions:**

Among the patients with normal LVEF and non-gender-related CHA_2_DS_2_-VA score 0~1 AF, the high H_2_FPEF score, and increasing age were independently associated with IS development (ClinicalTrials.gov Identifier: NCT02138695).

## Introduction

Atrial fibrillation (AF) is a significant risk factor for ischemic strokes (ISs), and the CHA_2_DS_2_-VASc score has been suggested to be the most reliable parameter for the IS risk stratification (1). The current guidelines recommend introducing oral anticoagulant therapy for stroke prevention in non-valvular AF patients with CHA_2_DS_2_-VASc scores of 2 points or higher. In contrast, no antithrombotic therapy is advantageous for men with scores of 0 or 1 point and women with scores of 1 or 2 points, respectively, in terms of the risk-benefit profile (2, 3). Nevertheless, AF patients with low CHA_2_DS_2_-VASc scores who are not recommended to undergo anticoagulant therapy exhibit an annual IS risk of 1.15% per year (4–7). This level of IS risk is similar to the annual IS risk in AF patients with higher CHA_2_DS_2_-VASc scores who are on anticoagulant therapy (5, 8). Therefore, identifying IS-related factors or clinical predictors of ISs in AF patients with low CHA_2_DS_2_-VASc scores might be valuable for stroke prevention in relatively young and active low-risk patients with AF (9–11). However, the mechanisms of IS in patients with AF are heterogeneous and the CHA_2_DS_2_-VASc score comprehensively evaluates both cardioembolic and non-cardioembolic risks, such as complex aortic plaque and carotid and intracranial arteriosclerosis (12). For this reason, IS risk-assessment studies in patients with low CHA_2_DS_2_-VASc scores and low numbers of comorbidities have been limited by epidemiologic data dependent on the International Classification of Diseases code (13). In this study, we explored the potential risk factors of ISs among patients with non-gender-related CHA_2_DS_2_-VA score 0~1 AF depending on the H_2_FPEF score (14), which is a recently developed risk score for heart failure with preserved ejection fraction (HFpEF) and known to be related with atrial myopathy (15). Additionally, we evaluated the outcome of AF catheter ablation (AFCA). The purpose of this study was to compare and assess the risk factors of ISs in the patients with non-gender-related CHA_2_DS_2_-VA score 0~1 AF.

## Materials and Methods

Data supporting the findings of this study are available from the corresponding author upon reasonable request.

### Study Subjects

The study is conducted in compliance with the ethical rules of the Declaration of Helsinki (2013) as a statement of ethical principles for medical research involving human subjects by The World Medical Association and approved by the Institutional Review Board of Yonsei University Health System. From January 2009 to April 2020, 1,353 patients with a diagnosis of AF were identified as having a normal left ventricular (LV) systolic function and low non-gender-related low CHA_2_DS_2_-VA score (0–1 points both in men and women) in the Yonsei AF Ablation Cohort Database (ClinicalTrials.gov Identifier: NCT02138695) and underwent AFCA for symptomatic and drug-refractory non-valvular AF. Written informed consent was obtained from all patients before the study inclusion. The exclusion criteria for AFCA were as follows: (1) permanent AF refractory to electrical cardioversion; (2) presence of a left atrial (LA) or LA appendage thrombus on transesophageal echocardiography; (3) no measurements of the left ventricular (LV) diameter, LV end-diastolic dimension (LVEDD), or ratio between the early mitral inflow velocity and mitral annular early diastolic velocity (EEm) by transthoracic echocardiography; (4) significant structural heart disease other than LV hypertrophy, such as significant valvular heart disease of grade two or greater, hypertrophic/ischemic/dilated cardiomyopathy, or congenital heart disease; (5) a history of a prior AFCA or cardiac surgery; and (6) a left ventricular ejection fraction (LVEF) ≤ 50%. Among those patients, 113 experienced IS despite non-gender-related CHA_2_DS_2_-VA score 0~1, and divided into groups at high risk of and medium/low risk of a cardioembolic stroke, respectively, based on the Trial of Org 10172 in Acute Stroke Treatment (TOAST) classification scheme (16) for the analysis of stroke subtype differences. All included patients underwent AFCA, and the time difference between the previous stroke event and AFCA and comorbidities was confirmed by the electrical medical record review. The CHA_2_DS_2_-VA score was recalculated immediately before the IS event by medical record review in patients with a prior IS.

### Calculating the H_2_FPEF Score

The H_2_FPEF score has six variables based on clinical and echocardiographic values: heaviness (body mass index (BMI) > 30 kg/m^2^, 2 points), hypertension (on two or more antihypertensive medicines, 1 point), atrial fibrillation (paroxysmal or persistent, 3 points), pulmonary hypertension (Doppler echocardiographic estimated pulmonary artery systolic pressure > 35 mmHg, 1 point), elderly status (age > 60 years, 1 point), and filling pressure (Doppler echocardiographic E/Em > 9, 1 point). The baseline H_2_FPEF score was calculated through medical record review same as the CHA_2_DS_2_-VA score recalculation in patients with a prior IS except echocardiographic parameters. In patients without a previous IS, the baseline H_2_FPEF score was calculated with the variables obtained within 3 months before the AFCA.

### Echocardiographic Measurement

Transthoracic echocardiography was conducted in all patients using commercially available devices (Vivid 7 or Vivid E9 from GE Healthcare, Chicago, IL, USA or iE 33 from Philips, Amsterdam, the Netherlands) as recommended by the American Society of Echocardiography (17). Standard images were obtained in the parasternal and apical views through two-dimensional, Doppler, and M-mode images, such as the LA anteroposterior diameter and LV end-systolic and LVEDD dimensions. The early Doppler mitral inflow (E) was recorded by the pulsed wave from the apical window, with a 1- to 3-mm pulsed Doppler sample volume placed between the tips and mitral leaflets during diastole. The early diastolic mitral annular velocity (Em) was recorded as the peak early diastolic tissue velocity using color Doppler tissue imaging of the septal mitral annulus. The early diastolic mitral inflow velocity ratio to the early diastolic mitral annular velocity (E/Em) was calculated. Tricuspid regurgitation (TR) and estimated right atrial (RA) pressure were evaluated using the recommended methods, and the right ventricular systolic pressure (RVSP) was calculated as 4 × (TR jet)^2^ + estimated RA pressure (18). For Doppler-derived parameters, at least 3 consecutive beats were measured and averaged (19).

### Electrophysiologic Characterization and Radiofrequency Catheter Ablation

Intracardiac electrograms were obtained using the Prucka CardioLab electrophysiology system (GE Healthcare, Chicago, IL, USA). A 3D electroanatomical map (Ensite NavX; Abbott Laboratories, Chicago, IL, USA; CARTO3; Johnson & Johnson Inc., NJ, USA.) was generated using a circumferential pulmonary-vein mapping catheter through a long sheath (Schwartz left 1; Abbott Laboratories, Chicago, IL, USA) through merging the 3D geometry generated by the electroanatomic mapping system with the corresponding 3D spiral CT images. Separately, a 3D LA voltage mapping was performed by obtaining the contact bipolar electrograms from 350 to 500 points on the LA endocardium during atrial pacing (high RA; pacing cycle length: 500 ms). The bipolar electrograms were filtered at 32–300 Hz. Color-coded voltage maps were generated by using the bipolar electrograms, and the peak-to-peak voltage was generated as previously described (20).

### Statistical Analysis

The baseline characteristics of patients were compared using descriptive statistics, presented as median (interquartile interval) values for continuous variables and as numbers (percentages) for categorical variables. With reference to a previous study (14), we set a 5 point H_2_FPEF score as the cut-off value, which has shown the probability of HFpEF >80%. To identify factors associated with the presence of a stroke, we performed univariate and multivariable logistic regression analyses. We conducted three models of multivariable logistic regression analyses because of the multicollinearity among the H_2_FPEF score and age or E/Em. Model 1 was analyzed by adding variables that were having *p* < 0.10 of the univariate models. Model 2 was analyzed by treating the H_2_FPEF score as a continuous variable, and model 3 was analyzed by treating it as a categorical variable. To compare the effect of individual H_2_FPEF score variables, we performed multivariable logistic regression based on individual H_2_FPEF score variables. A subgroup analysis was performed based on the comorbidities not included in the H_2_FPEF score variables. Two-sided *p*-values of <0.05 were considered to be statistically significant. Statistical analyses were conducted using SAS version 9.4 (SAS Institute, Cary, NC, USA) and R version 4.0.0 (R Foundation for Statistical Computing, Vienna, Austria) software.

## Results

### Baseline Patient Characteristics

Among 3,648 consecutive patients in this single-center prospective registry, we included 1,353 patients with AF (24.7% female, median age 56 years, paroxysmal AF 72.6%) who had non-gender-related CHA_2_DS_2_-VA score 0~1 at the times of enrollment (*n* = 1,240) or previous IS events (*n* = 113), normal LV systolic function, and available H_2_FPEF score. The time difference between the previous stroke events and inclusion was a median of 1.0 [interquartile range (IQR):1.0–4.0] year in 113 patients with previous stroke events, and 99 of them had a high risk for cardioembolism to the TOAST classification. About 84% of previous stroke events (95/113) had occurred within a year.

### Factors Associated With ISs in Patients With Non-Gender-Related CHA_2_DS_2_-VA Score 0~1

We compared the AF patients with non-gender-related CHA_2_DS_2_-VA score 0~1 and those at the time of the IS in Table 1. Patients who experienced IS at the time of non-gender-related CHA_2_DS_2_-VA score 0~1 were older (*p* < 0.001) and had a higher baseline H_2_FPEF score (*p* = 0.018), E/Em values (*p* < 0.001), and RVSP (*p* = 0.007), larger LA dimension (*p* = 0.003), lower eGFR (*p* = 0.001), and left atrium appendage (LAA) peak velocity (*p* < 0.001) than those without IS.

**Table 1:** Comparison of the baseline characteristics between non-gender-related CHA_2_DS_2_-VA score 0~1 AF patients with strokes and those without strokes.

**Variables**	**Overall, *N* = 1,353**	**Low CHA_**2**_DS_**2**_-VA prior stroke (+), *N* = 113**	**Low CHA_**2**_DS_**2**_-VA prior stroke (–), *N* = 1,240**	***p*-value**
Age (years)	56 (50, 62)	62 (58, 67)	56 (49, 61)	<0.001
Female, *n* (%)	334 (24.7)	28 (24.8)	306 (24.7)	1.000
Smoking, *n* (%)	513 (38.0)	45 (39.8)	468 (37.8)	0.104
Alcohol, *n* (%)	688 (50.9)	52 (46.0)	636 (51.3)	0.508
Paroxysmal AF, *n* (%)	978 (72.6)	77 (68.1)	901 (73.0)	0.317
Heart failure, *n* (%)^*^	35 (2.6)	5 (4.4)	30 (2.4)	0.329
Hypertension, *n* (%)	383 (28.3)	31 (27.4)	352 (28.4)	0.915
Diabetes, *n* (%)	44 (3.3)	6 (5.3)	38 (3.1)	0.312
Vascular disease, *n* (%)	22 (1.6)	4 (3.5)	18 (1.5)	0.196
BMI (kg/m^2^)	24.7 (22.9, 26.6)	24.3 (23.1, 26.1)	24.7 (22.9, 26.6)	0.252
H_2_FPEF score, baseline	5 (4, 6)	6 (4, 6)	5 (4, 6)	0.018
eGFR, (mL/min/1.73 m^2^)	89 (78, 103)	84 (74, 94)	90 (79, 103)	0.001
**Medication**				
Beta blocker, *n* (%)	493 (35.0)	42 (37.2)	431 (34.8)	0.685
ACEi/ARB, *n* (%)	283 (20.9)	22 (19.5)	261 (21.1)	0.781
Statin, *n* (%)	324 (24.0)	62 (54.9)	262 (21.1)	<0.001
**Echocardiography**				
LA diameter (mm)	40 (36, 44)	42 (39, 45)	40 (36, 44)	0.001
LVEF (%)	64 (60, 69)	63 (59, 67)	65 (60, 69)	0.017
E/Em	8.5 (7.0, 11.0)	10.0 (8.0, 12.1)	8.4 (7.0, 10.7)	<0.001
DT (ms)	175 (155, 205)	171 (153, 208)	177 (155, 205)	0.444
TR jet (m/s)	2.2 (2.0, 2.4)	2.3 (2.1, 2.5)	2.2 (2.0, 2.4)	0.006
RVSP (mmHg)	25 (22, 28)	26 (23, 29)	25 (21, 28)	0.007
LAA peak velocity (cm/s^†^	49 (34, 67)	37 (25, 58)	51 (36, 67)	<0.001
**LA pressure (mmHg)**				
Peak	21 (15, 27)	20 (14, 27)	21 (15, 27)	0.423
Mean	11 (8, 16)	11 (7, 15)	11 (8, 16)	0.430
Nadir	4 (0, 8)	4 (1, 7)	4 (0, 8)	0.983
**3D bipolar mean voltage (mV)**				
LA voltage	1.4 (0.9, 1.9)	1.3 (0.8, 1.7)	1.4 (0.9, 1.9)	0.161
LAA voltage	2.2 (1.3, 3.3)	2.1 (1.2, 3.1)	2.3 (1.3, 3.3)	0.261

*The data are presented as the number (%), and median (interquartile interval). Non-parametric continuous variables as assessed by the Kolmogorov–Smirnov method, were analyzed by Kruskal–Wallis H test*.

*ACEi, angiotensin-converting-enzyme inhibitor; AF, atrial fibrillation; ARB, angiotensin receptor blocker; BMI, body mass index; DT, deceleration time; E/Em, the ratio of the early diastolic mitral inflow velocity (E) to the early diastolic mitral annular velocity (Em); GFR, glomerular filtration rate; LA, left atrium; LAA, left atrium appendage; LVEF, left ventricular ejection fraction; RVSP, right ventricular systolic pressure; TR, tricuspid regurgitation*.

* *Defined as conventional HFpEF diagnosis criteria: left ventricular ejection fraction ≥50% with exertional dyspnea that was not caused by extracardiac causes*.

† *Velocity was measured at transesophageal echocardiography*.

### LV Diastolic Dysfunction and IS at Non-Gender-Related CHA_2_DS_2_-VA Score 0~1

**Figure 1:**
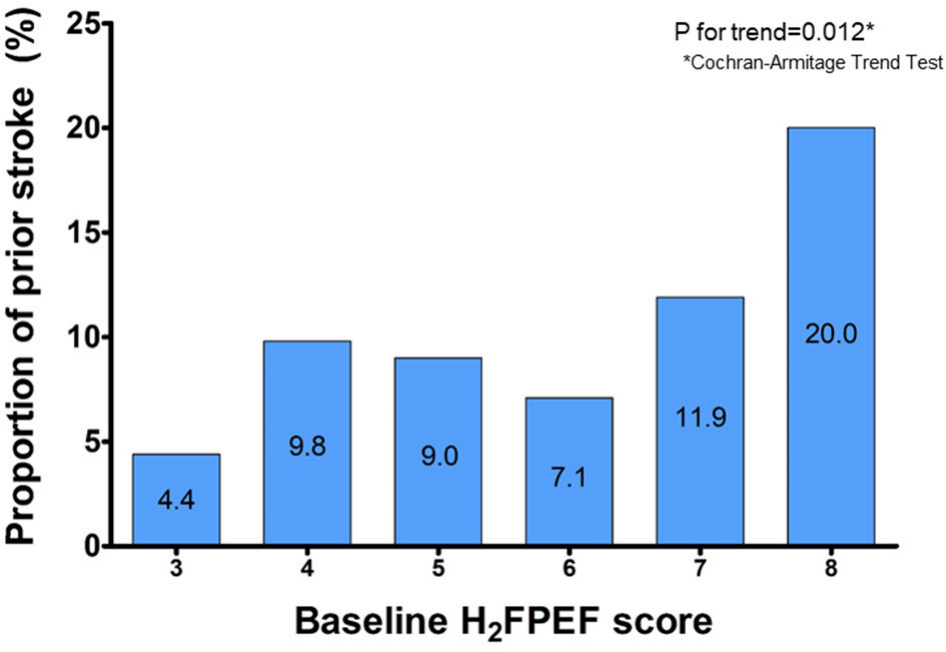
The proportion of prior stroke according to the baseline H_2_FPEF scores.

Figure 1 shows a linear relationship trend between the baseline H_2_FPEF score and non-gender-related CHA_2_DS_2_-VA score 0~1. Patients with the baseline H_2_FPEF score ≥ 5 were generally older (*p* < 0.001) and had higher proportions of hypertension (*p* < 0.001). They had an increased BMI value (*p* < 0.001), larger LA diameters (*p* < 0.001), higher E/Em values (*p* < 0.001) and RVSP (*p* < 0.002), and higher prescription rate of renin-angiotensin-aldosterone system blockers (*p* < 0.001) and statins (*p* = 0.004, Table 2).

**Table 2:** Comparison of the baseline characteristics based on H_2_FPEF score 5 in non-gender-related CHA_2_DS_2_-VA score 0~1 patients with AF.

**Variables**	**H**_**2**_ **FPEF score, baseline**		
	** <5 (*n* = 566)**	**≥5 (*n* = 787)**	***p*-value**
Age (years)	55 (50, 60)	58 (50, 63)	<0.001
Female, *n* (%)	146 (25.8)	188 (23.9)	0.460
Smoking, *n* (%)	215 (38.0)	298 (37.9)	0.858
Alcohol, *n* (%)	281 (49.7)	407 (51.8)	0.642
Paroxysmal AF, *n* (%)	420 (74.6)	558 (71.2)	0.184
Heart failure, *n* (%)^*^	15 (2.7)	20 (2.5)	1.000
Hypertension, *n* (%)	106 (18.7)	277 (35.2)	<0.001
Diabetes, *n* (%)	19 (3.4)	25 (3.2)	0.977
Vascular disease, *n* (%)	8 (1.4)	14 (1.8)	0.759
Prior stroke, *n* (%)	40 (7.1)	73 (9.3)	0.177
BMI (kg/m^2^)	23.2 (21.7, 24.2)	26.2 (25.1, 27.7)	<0.001
eGFR (mL/min)	92 (80, 104)	88 (77, 101)	0.021
**Medication**
Beta blocker, *n* (%)	181 (32.0)	292 (37.2)	0.056
ACEi/ARB, *n* (%)	68 (12.0)	215 (27.4)	<0.001
Statin, *n* (%)	113 (20.0)	211 (26.8)	0.004
**Echocardiography**
LA diameter (mm)	38 (35, 42)	41 (37, 45)	<0.001
LVEF (%)	64 (60, 69)	65 (60, 69)	0.960
EEm	7.9 (6.7, 9.0)	9.4 (8.0, 11.8)	<0.001
DT (ms)	179 (157, 206)	175 (154, 204)	0.330
TR jet (m/s)	2.2 (2.0, 2.4)	2.2 (2.0, 2.4)	<0.001
RVSP (mmHg)	24 (21, 28)	25 (22, 29)	0.002
LAA peak velocity (cm/s)^†^	48 (36, 67)	50 (33, 67)	0.550
**LA pressure (mmHg)**
Peak	21 (16, 27)	20 (15, 27)	0.255
Mean	12 (8, 16)	11 (8, 16)	0.256
Nadir	4 (1, 8)	4 (0, 8)	0.471
**3D bipolar mean voltage (mV)**
LA voltage	1.4 (0.9, 1.8)	1.4 (0.9, 1.9)	0.903
LAA voltage	2.3 (1.4, 3.3)	2.2 (1.3, 3.3)	0.469

*The data are presented as the number (%), and median (interquartile interval). Non-parametric continuous variables as assessed by the Kolmogorov-Smirnov method were analyzed by the Kruskal-Wallis H test*.

*ACEi, angiotensin-converting-enzyme inhibitor; AF, atrial fibrillation; ARB, angiotensin receptor blocker; BMI, body mass index; DT, deceleration time; E/Em, the ratio of the early diastolic mitral inflow velocity (E) to the early diastolic mitral annular velocity (Em); GFR, glomerular filtration rate; LA, left atrium; LAA, left atrium appendage; LVEF, left ventricular ejection fraction; RVSP, right ventricular systolic pressure; TR, tricuspid regurgitation*.

* *Defined as conventional HFpEF diagnosis criteria: left ventricular ejection fraction ≥50% with exertional dyspnea that was not caused by extracardiac causes*.

† *Velocity was measured at transesophageal echocardiography*.

The univariate and multivariate analysis for IS in patients with non-gender-related CHA_2_DS_2_-VA score 0~1 is listed in Table 3. For the multivariate logistic regression analyses, we tested 2 models because of collinearity between age and H_2_FPEF score. Age [*OR* 1.11 (1.07–1.17), *p* < 0.001, Model 1] and H_2_FPEF score as continuous [*OR* 1.31 (1.03–1.67), *p* = 0.028, Model 2] variables were independently associated with ISs by multivariate analysis. The eGFR was also independently associated with IS at low CHA_2_DS_2_-VA scores in both Models 1 and 2.

**Table 3:** Univariate and multivariate logistic regression analysis for the predictors of prior ISs in patients with non-gender-related CHA_2_DS_2_-VA score 0~1 AF.

	**Univariate analysis**		**Multivariable model 1^†^**		**Multivariable model 2^‡^**	
	**Unadjusted OR (95% CI)**	***p*-value**	**Adjusted OR (95% CI)**	***p*-value**	**Adjusted OR (95% CI)**	***p*-value**
Age	1.10 (1.08–1.14)	<0.001	1.11 (1.07–1.17)	<0.001		
Female	1.01 (0.63–1.55)	0.981				
Smoking	1.09 (0.73–1.61)	0.672				
Alcohol	0.81 (0.55–1.19)	0.280				
Paroxysmal AF	0.79 (0.53–1.21)	0.267				
Heart failure^*^	1.87 (0.63–4.52)	0.206				
Hypertension	0.95 (0.61–1.45)	0.829				
Diabetes	1.77 (0.66–4.00)	0.204				
Vascular disease	2.49 (0.71–6.82)	0.104				
BMI	0.96 (0.90–1.02)	0.203				
H_2_FPEF score, baseline (continuous variable)	1.21 (1.05–1.40)	0.008			1.31 (1.03–1.67)	0.028
eGFR	0.98 (0.97–0.99)	0.002	0.98 (0.96–1.00)	0.021	0.98 (0.96–0.99)	0.004
**Echocardiography**
LA diameter	1.06 (1.03–1.10)	0.001	0.97 (0.92–1.03)	0.387	0.98 (0.93–1.04)	0.575
LVEF	0.96 (0.93–0.99)	0.023	0.93 (0.88–0.98)	0.013	0.96 (0.91–1.02)	0.408
E/Em	1.09 (1.05–1.13)	<0.001	1.12 (1.04–1.19)	0.001		
DT	1.00 (0.99–1.00)	0.604				
TR jet	1.13 (0.86–1.45)	0.243				
RVSP	1.05 (1.02–1.08)	0.001	0.99 (0.94–1.05)	0.823		
LAA peak velocity	0.98 (0.97–0.99)	0.001	1.00 (0.99–1.02)	0.906	0.99 (0.98–1.01)	0.296
**LA pressure**
Peak	1.00 (0.97–1.02)	0.684				
Mean	1.00 (0.97–1.03)	0.902				
Nadir	1.00 (0.97–1.04)	0.702				
**3D bipolar mean voltage**
LA voltage	0.98 (0.58–1.64)	0.935	0.98 (0.58–1.64)	0.935	0.78 (0.47–1.26)	0.321
LAA voltage	0.90 (0.75–1.07)	0.254				

*CI, confidence interval; OR, Odds ratio*.

*AF, atrial fibrillation; BMI, body mass index; DT, deceleration time; E/Em, the ratio of the early diastolic mitral inflow velocity (E) to the early diastolic mitral annular velocity (Em); GFR, glomerular filtration rate; LA, left atrium; LAA, left atrium appendage; LVEF, left ventricular ejection fraction; RVSP, right ventricular systolic pressure; TR, tricuspid regurgitation*.

* *Defined as conventional HFpEF diagnosis criteria: LVEF ≥ 50% with exertional dyspnea that was not caused by extracardiac causes*.

† *Model 1: age, eGFR, LA diameter, LVEF, E/Em, RVSP, LAA peak velocity, and LA mean voltage*.

‡ *Model 2: H2FPEF score (continuous variable), eGFR, LA diameter, LVEF, LAA peak velocity, and LA mean voltage*.

### The Effect of Individual Factors of H_2_FPEF Score and Subgroup Analysis

**Figure 2:**
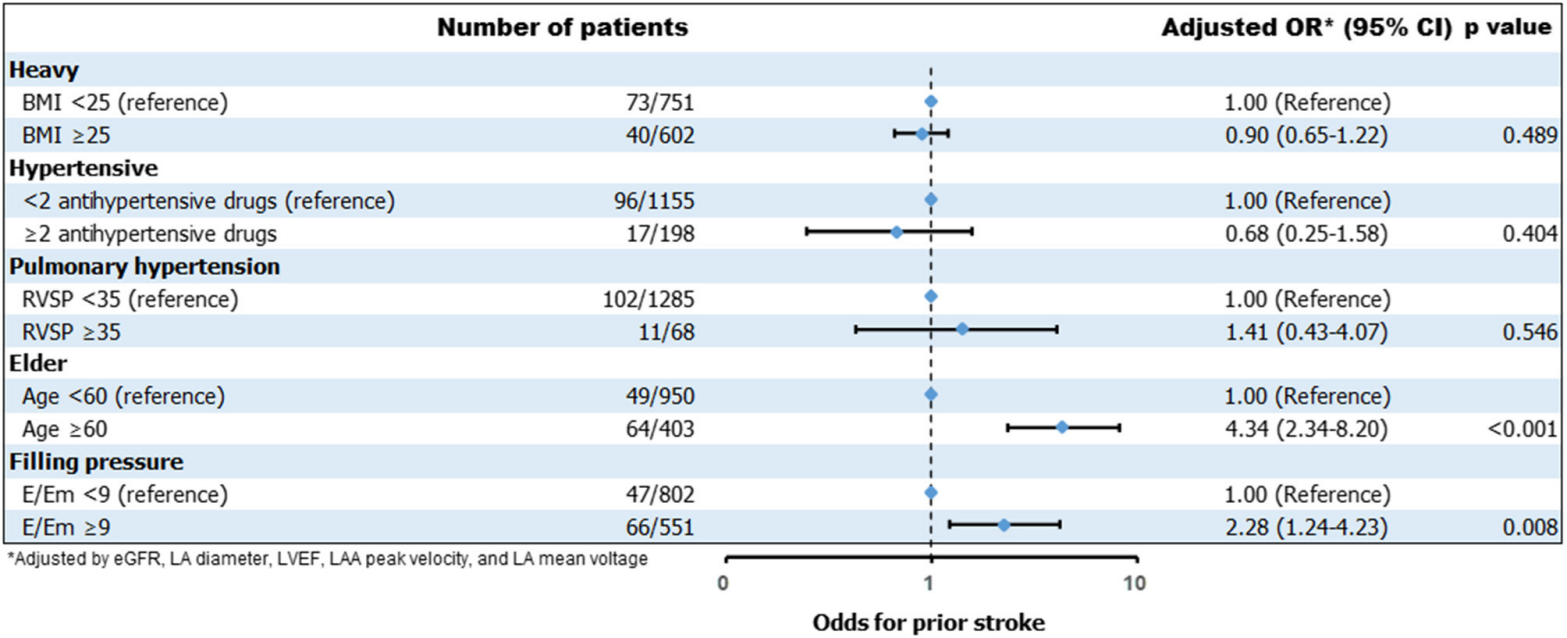
Odds for prior stroke based on the individual factors of H_2_FPEF score.

**Figure 3:**
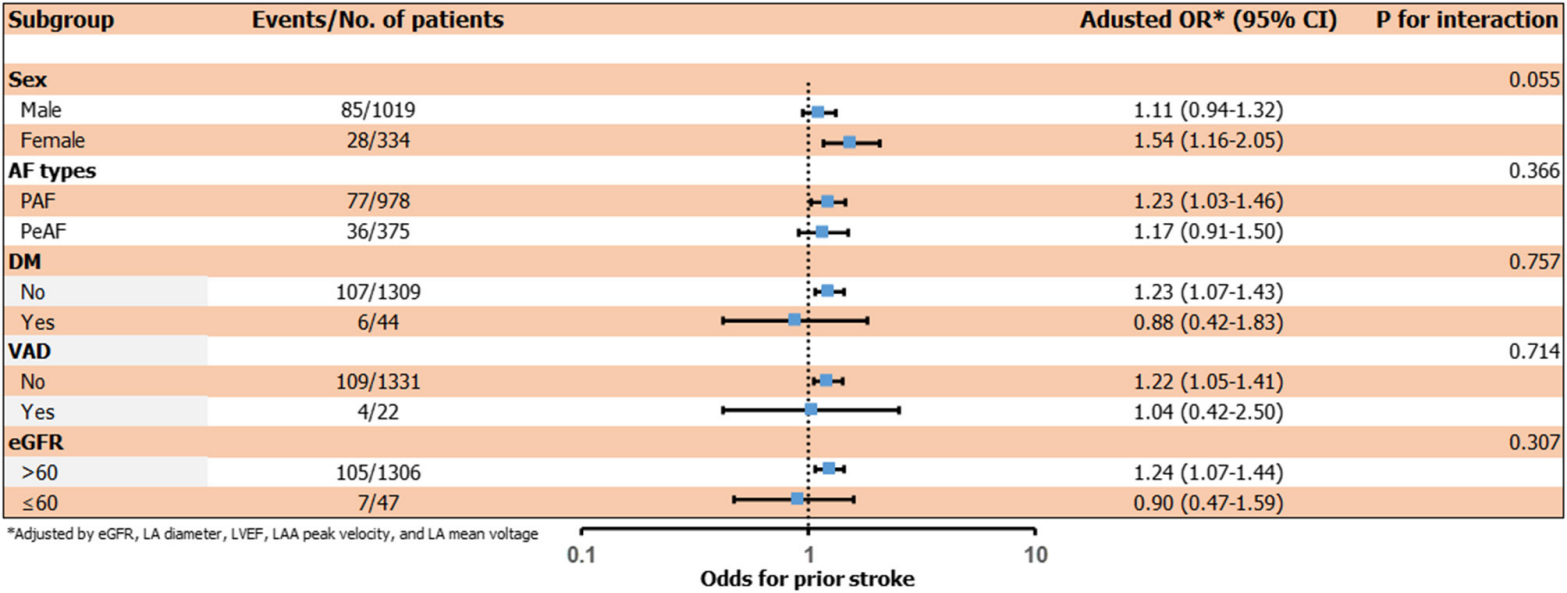
Subgroup analysis of the risk of prior stroke by an increase in the H_2_FPEF score.

We compared the effect of individual variables of the H_2_FPEF score on IS at non-gender-related CHA_2_DS_2_-VA score 0~1 in the logistic regression models (Figure 2). Among six variables, age ≥60 years [*OR* 4.34 (2.34–8.20), *p* < 0.001] and E/Em ≥9 [*OR* 2.28 (1.24–4.23), *p* = 0.008] were independently associated with ISs in this low-risk group. In the subgroup analysis, the H_2_FPEF score was consistently related to the risk of ISs at non-gender-related CHA_2_DS_2_-VA score 0~1, regardless of sex, AF types, diabetes, vascular disease, and renal function (Figure 3).

### Rhythm Outcome of AFCA

**Figure 4:**
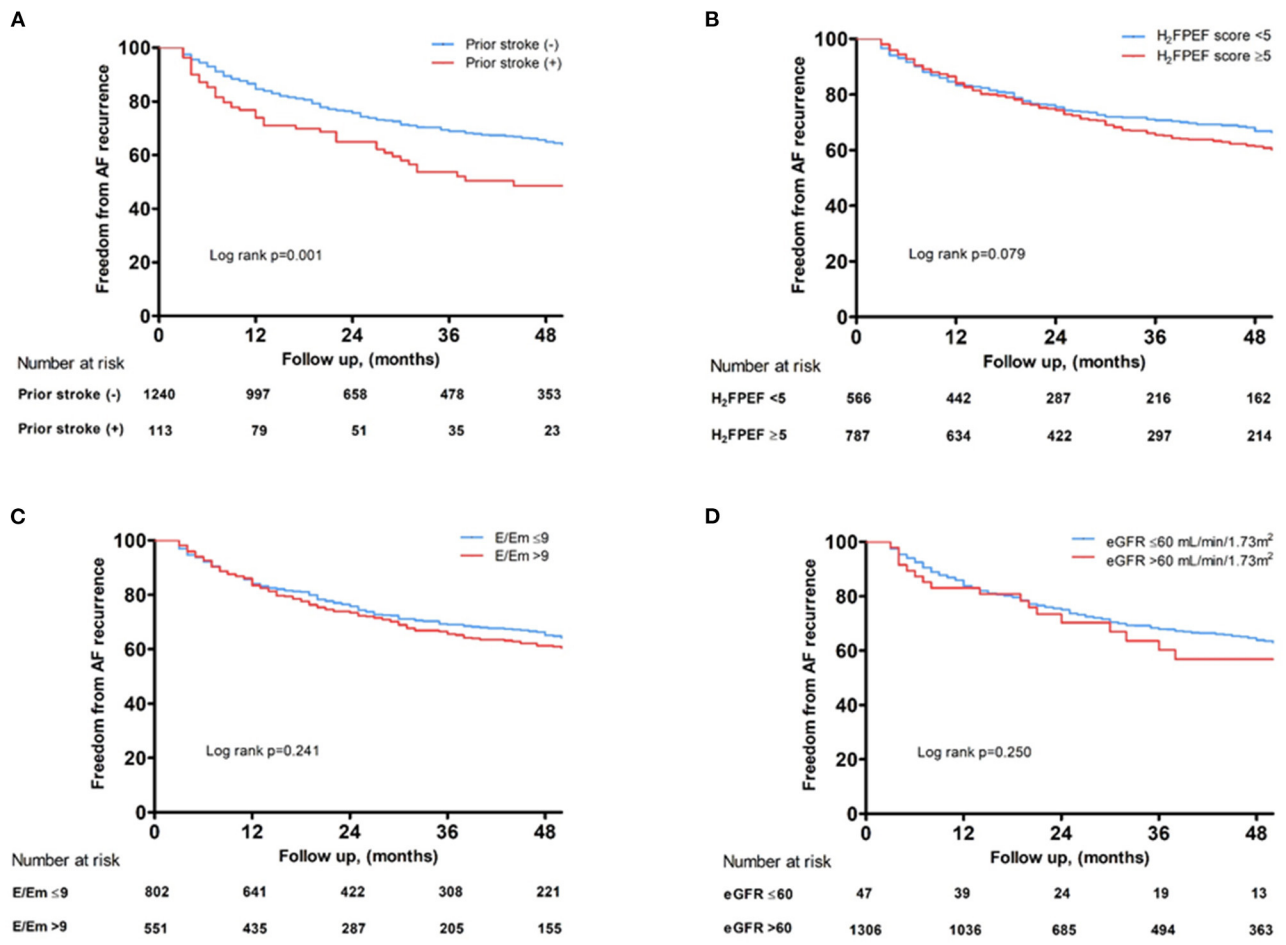
Kaplan–Meier curves showing the rate of freedom from AF recurrence after AFCA depending on a prior ISs **(A)**, baseline H_2_FPEF score **(B)**, E/Em **(C)**, and renal function **(D)**. AF, atrial fibrillation; AFCA, atrial fibrillation catheter ablation; E/Em, the ratio of the early diastolic mitral inflow velocity (E) to the early diastolic mitral annular velocity (Em); IS, ischemic stroke.

Over median 28 months of follow-up, the cumulative AF recurrence rate after AFCA was significantly higher in the patients with IS under non-gender-related CHA_2_DS_2_-VA score 0~1 (log-rank *p* = 0.001, Figure 4A), but not high H_2_FPEF score ≥ 5 (log-rank *p* = 0.079, Figure 4B), E/Em > 9 (log-rank *p* = 0.241, Figure 4C), or eGFR ≤ 60 ml/min/1.73 m^2^ (log-rank *p* = 0.250, Figure 4D).

## Discussion

### Main Findings

This study explored the risk factors for ISs based on the H_2_FPEF score in AF patients with non-gender-related CHA_2_DS_2_-VA score 0~1. Among these low-risk patient groups, increased age, the high H_2_FPEF score, and low eGFR were independently associated with ISs. Among six variables of the H_2_FPEF score, age over 60 years, and E/Em ≥ 9 showed a significantly greater risk of ISs at non-gender-related CHA_2_DS_2_-VA score 0~1 among the patients with AF who were referred for catheter ablation.

### CHA_2_DS_2_-VASc Score and Low IS Risk Patients With AF

We have been using the CHA_2_DS_2_-VASc score as an epidemiologically reasonable stroke prevention index (21). In spite of the 1.15% annual risk of ISs, the existing guidelines do not recommend antithrombotic therapy to AF patients with non-gender related CH_2_DS_2_-VASc scores of <2-point ( ≤ 1 point in men and ≤ 2 points in women) (2, 3, 5–7). However, it remains clinically important to predict and prevent ISs in these low-risk patients, primarily young and active individuals. Another weak point is that the CH_2_DS_2_-VASc score components include not only cardioembolic but also non-cardioembolic risk factors (12) and do not encompass pathophysiological mechanisms, such as atrial myopathy, hemodynamic factors, the AF burden, or hypercoagulability (22, 23).

### H_2_FPEF Score and Risk of ISs in AF Patients With Low CHA_2_DS_2_-VASc Scores

Although the associated comorbidities tend to be less, hemodynamic factors are more likely to contribute to the mechanism of IS in AF patients with non-gender-related CHA_2_DS_2_-VA score 0~1 in this study. Recently, growing interest in the potential for an atrial myopathy that leads to AF progression and contributes to systemic thromboembolism has emerged (24). King et al. (25) discerned the relationship between atrial fibrosis and the risk of a stroke in patients with AF using late-gadolinium enhanced cardiac MRI. Leong et al. (23) reported LA dysfunction contributes to the mechanism of ISs by analyzing the LA strain. Atrial myopathy generates the condition vulnerable to atrial dysfunction, fibrosis, structural remodeling, and blood stasis, increasing the risk of thrombus development.

Although genetic factors may contribute to atrial myopathy in certain specific low-risk patients with AF (26, 27), the LV diastolic dysfunction could be a key contributing factor. LV diastolic dysfunction increases the atrial filling pressures and triggers progressive atrial enlargement, dysfunction, and atrial myopathy, eventually leading to ISs (28). Kim et al. (29) and Yu et al. (30) reported that LV diastolic dysfunction represented by the E/Em is associated with a greater risk for ISs and LA remodeling, especially in female patients with AF. The recently developed integrated scoring system, the H_2_FPEF score (14), which estimates an adverse effect on hemodynamics, may help to identify LA myopathy (15, 31). Furthermore, we proved the baseline H_2_FPEF score is independently associated with ISs in AF patients with non-gender-related CHA_2_DS_2_-VA score 0~1.

### Clinical Implications

Based on the results of this study, physicians should consider the potential risk of ISs in AF patients with non-gender-related CHA_2_DS_2_-VA score 0~1, especially in patients with increased age, a high H_2_FPEF score, or renal dysfunction. Recently, we demonstrated that the active rhythm control of AF by AFCA is superior to medical therapy in the risk reduction of ISs (32) and AFCA reduces the H_2_FPEF score a year after the procedure in AF patients with underlying LV diastolic dysfunction (33). Therefore, despite a non-gender-related CHA_2_DS_2_-VA score of 0~1, we have to pay more attention to the risk of IS or rhythm control status for those patients with old age, the high H_2_FPEF score, or renal dysfunction.

### Limitations

Our study had several limitations that should be noted. First, the population of this study was a single-center AFCA cohort with detailed clinical and imaging data. As these patients were referred for AFCA, the results of this study cannot be generalized. Second, ISs were classified and diagnosed by neurologists, and silent ISs were not excluded in the study. Third, because this study is retrospectively designed including the AF population with detailed imaging and physiological data, the timing of the IS event was not accurately reflected in the baseline characteristics. However, most of the prior strokes (84%) occurred within 1 year of the study time point. Furthermore, we checked precise age at the time of stroke events in patients with ISs and adjusted the non-gender-related CHA_2_DS_2_-VA score. Fourth, although their proportion was small, we did not exclude the patients with mitral annular calcification or mitral regurgitation whose E/Em could not represent LV filling pressure.

## Conclusion

Among AF patients with normal LV systolic function and non-gender-related CHA_2_DS_2_-VA score 0~1, age, renal function, and the high H_2_FPEF score, that may allow for LA myopathy identification, were significantly associated with the ISs.

## Data Availability Statement

The raw data supporting the conclusions of this article will be made available by the authors, without undue reservation.

## Ethics Statement

The studies involving human participants were reviewed and approved by Institutional Review Board of Yonsei University Health System. The patients/participants provided their written informed consent to participate in this study.

## Author Contributions

H-NP and MK: conceptualization, validation, writing—original draft, and writing—review and editing. H-NP, HY, T-HK, J-SU, YK, HN, BJ, M-HL, and JH: data curation. MK: formal analysis and Software. H-NP, MK, HY, T-HK, D-IL, BJ, and M-HL: methodology. H-NP, MK, HY, T-HK, D-IL, J-SU, YK, HN, BJ, M-HL, and JH: investigation. All authors contributed to the article and approved the submitted version.

## Funding

This work was supported by grants (HI19C0114 and HI21C0011) from the Ministry of Health and Welfare and a grant (NRF-2020R1A2B01001695) from the Basic Science Research Program run by the National Research Foundation of Korea (NRF), which was funded by the Ministry of Science, ICT & Future Planning (MSIP).

## Conflict of Interest

The authors declare that the research was conducted in the absence of any commercial or financial relationships that could be construed as a potential conflict of interest.

## Publisher's Note

All claims expressed in this article are solely those of the authors and do not necessarily represent those of their affiliated organizations, or those of the publisher, the editors and the reviewers. Any product that may be evaluated in this article, or claim that may be made by its manufacturer, is not guaranteed or endorsed by the publisher.
